# Fracture Resistance of Thermal‐Mechanical Aged Resin Composite‐Restored Premolars: Effect of Different Application Protocols of a Universal Adhesive

**DOI:** 10.1002/cre2.70424

**Published:** 2026-08-19

**Authors:** Fereshteh Shafiei, Marzieh Moradian, Marziyeh Naseri Mojarad

**Affiliations:** ^1^ Oral and Dental Disease Research Center, Department of Operative Dentistry, School of Dentistry Shiraz University of Medical Sciences Shiraz Iran; ^2^ Student Research Committee, Department of Operative Dentistry, School of Dentistry Shiraz University of Medical Sciences Shiraz Iran

**Keywords:** acid etching, fracture strength, resins, universal adhesive

## Abstract

**Objectives:**

This research investigated the effect of different applications of Universal bonding on the fracture resistance (FR) of maxillary premolars restored with mesio‐occluso‐distal resin composite.

**Materials and Methods:**

One‐hundred and eight extracted maxillary premolars were randomly assigned into nine groups (*n* = 12): (1) ST, sound teeth; (2) nonrestored teeth; (3) SEE, selective enamel etching; (4) E&R, etch‐and‐rinse; (5) SDE, selective dentin etching; (6) SEE+resin layer; (7) E&R+resin layer; (8) SDE+resin layer/precuring; (9) SDE+resin layer/co‐curing. Following restoration, mechanical loading (100,000 cycles, 50 N), 6‐month water storage, and thermocycling (1000 cycles), FR was tested. Failure mode was evaluated. Data were analyzed using one‐way ANOVA and Tukey test (*α* = 0.05).

**Results:**

SDE+resin layer/precuring showed the highest mean fracture load (1222.0 ± 58.7 N), with a significant difference with all groups (*p* < 0.001), except SDE+resin layer/co‐curing (1166.5 ± 50.3 N). FR of SEE with the lowest value (894.50 ± 70.41 N) was lower than SDE (*p* < 0.001) but comparable with E&R. Resin layer significantly improved FR in all etching conditions, except the E&R group (*p* = 0.272). The highest number of restorable fractures was in two SDE with resin layer groups.

**Conclusion:**

Adhesive application protocols affected FR. Combining short‐time dentin etching with a HEMA‐free hydrophobic resin may be suggested to increase the stable reinforcement potential of universal adhesive‐resin composite restorations in premolars and preserve teeth under high loading.

**Clinical Relevance:**

The adhesive application protocol considerably affected the fracture resistance of MOD resin composite–restored premolars. A short time dentin etching using an ultra‐mild universal adhesive, along with increasing hydrophobicity of the adhesive interface, may enhance the mechanical reinforcement of resin composite‐restored premolars during simulated thermal‐mechanical intraoral conditions. This approach also possibly prevents the risk of catastrophic failure under unexpectedly high loads.

## 1 Introduction

Cavity preparation for caries removal, along with removal of marginal ridges, may compromise the integrity of tooth structure and decrease fracture resistance (FR) (Camacho et al. 2007; Forster et al. 2019). In addition to re‐establishing esthetics and function, restorative procedures should be capable of recovering the lost strength of prepared teeth (Shafiei et al. 2019a). Composite resins used with adhesive systems can partially or completely meet these requirements and are therefore commonly employed in dental practice (Ferracane 2008; Shafiei et al. 2019a). Although uncontrolled polymerization shrinkage stress of composite resin may negatively affect the adhesive interface, the effective bonding performance of the adhesive protocol plays a critical role in the reinforcing capability of adhesive restorations (Ferracane 2008; Thongbai‐on et al. 2019).

Universal adhesives (UAs), introduced in 2011, represent a significant evolution in adhesive systems with multi‐mode application—self‐etch (SE), etch‐and‐rinse (ER), or selective enamel etching (SEE). Along with their simplified procedure, this versatility makes them attractive for clinicians (Chen et al. 2022; Cuevas‐Suarez et al. 2019). UAs essentially belong to one‐step self‐etch adhesive systems, in which acidic primers and adhesive resin are combined in a single bottle (Chen et al. 2022). Based on their pH, UAs can be categorized into ultra‐mild (pH > 2.5), mild (pH ≈ 2), and intermediately/strong (pH ≈ 1.5) (Van Meerbeek et al. 2020). Their pH appears to affect clinical effectiveness, with milder pH showing more desirable/effective results on dentin surfaces (Cuevas‐Suarez et al. 2019). Incorporation of functional monomers—mostly 10‐MDP—in UAs enables chemical interaction with hydroxyapatite crystals surrounding collagen fibrils in unetched dentin. The resulting Ca‐10‐MDP salts, known as nano‐layering, are resistant to hydrolysis and help stabilize the long‐term adhesive interface (Chen et al. 2022; Yoshida et al. 2012).

Two major concerns have been raised regarding UAs. The first is the potential interference of the smear layer created during cavity preparation with burs (Ahmed et al. 2019). Mild or ultra‐mild UAs may be unable to effectively etch through or adequately dissolve the smear layer, impairing their bonding performance (Stape et al. 2018). This issue is more relevant for the thicker, denser smear layer produced by burs in clinical practice compared to the lighter smear layer generated using 600‐grit SiC paper in laboratory studies (Stape et al. 2022, 2021, 2018). Residual smear layer debris at the bonded interface may also hinder polymer chain movement during polymerization (Muñoz et al. 2014). In the E&R approach, 15‐s phosphoric acid etching removes the smear layer and demineralizes the underlying dentin; however, incomplete penetration of resin into the entire depth of demineralized dentin may compromise bond strength (Pashley et al. 2011). To address this issue, some authors recommend selective dentin etching (SDE) between self‐etch (SE) and E&R mode. In this technique, a short time, 3–5 s acid etching removes the smear layer without overexposing collagen fibrils, reducing the risk of hydrolytic degradation by endogenous proteases. Moreover, preservation of hydroxyapatite in dentin could support chemical bonding with the MDP monomer in UAs (Stape et al. 2022, 2021, 2018). This resulted in a higher bond strength compared to SE mode with no alteration in Ca content of dentin (Stape et al. 2018). However, a recent study indicated no preference for SDE over SE and E&R mode for three tested UAs in terms of bond durability (Balaban and Arısu 2025).

UAs contain more solvent and less resin compared to systems where primer and adhesive are applied separately. This, along with their typically thin adhesive film (often 10–15 μm, or even < 10 μm), may cause suboptimal polymerization/stabilization due to the formation of an oxygen‐inhibition layer (Nunes et al. 2005; Van Meerbeek et al. 2020). This is the second concern associated with UAs (Ahmed et al. 2019). Based on the more hydrophilic nature and water absorption of UAs, some authors have suggested applying an additional hydrophobic adhesive (AHA) layer. (Throughout this article, the term resin layer refers to the AHA layer.) This protocol could compensate these negative phenomena on hydrolytically resistance (Ermis et al. 2019; Muñoz et al. 2014; Sezinando et al. 2015). However, conflicting results have been reported regarding the effect of AHA on the bonding performance of UAs, depending on the specific adhesive formulation and etching mode (Ahmed et al. 2019; Saitamon et al. 2024; Wang et al. 2023).

Recently, etching mode (SE and E&R) was reported to significantly affect the ability of UAs to provide high and stable fracture resistance (FR) of premolars with mesio‐occluso‐distal (MOD) resin composite restorations (Saitamon et al. 2024). However, the effect of SDE and resin layer, and their combination on the reinforcing ability of UA/resin composite restorations in teeth with MOD cavity have not yet been evaluated. Given the critical role of adhesive performance in reinforcing restored teeth, this study evaluated the effects of different application protocols of a UA on the FR of premolars with MOD resin composite restorations. The null hypothesis was that neither the etching mode (E&R, SEE, or SDE) of All‐Bond Universal (ABU) nor the use of an additional hydrophobic adhesive (AHA) layer after each etching mode would significantly affect the fracture resistance of resin composite–restored maxillary premolars after thermo‐mechanical aging.

## 2 Material and Method

Approval of the research protocol was obtained from the local academic ethics committee (Approval ID: IR.SUMS.DENTAL.REC.1403.072). Informed consent was obtained from all patients whose extracted teeth were used in the research.

### 2.1 Power Analysis

Based on a similar study (Saitamon et al. 2024), the effect size was calculated as *f* = 4.2. For this research, the sample size was determined using the lowest level of a large effect size (*f* ≥ 0.4), assuming a Type I error rate (*α*) of 0.05 and a Type II error rate (*β*) of 0.2. Accordingly, the required sample size was 12 specimens per group. Sample size estimation was performed using G*Power version 3.1.

### 2.2 Materials

The materials used in this study, along with their compositions and manufacturers’ instructions, are presented in Table 1.

**Table 1:** Materials used in the study, their compositions, and manufacturers’ instructions.

Manufacturer/Lot number	Material	Chemical compositions	Manufacture instructions
AllBond Universal (ABU), Bisco, Schaumburg, IL, USA/B7204	Universal adhesive	MDP, Bis‐GMA, HEMA, ethanol, water, initiator	Apply two separate coats of adhesive with scrubbing for 10–15 s per coat. Do not light cure between coats. Evaporate excess solvent by thoroughly air‐drying for at least 10 s. Light cure for 10 s.
Porcelain Bond (Bisco, Shaumburg, USA)/2400002834	Porcelain bonding resin	Bis‐GMA, TEGDMA, UDMA	Apply a thin layer to the surface using a microbrush. Air‐dry gently for 5 s and light‐cure for 10 s.
Filtek Z250 XT, 3M ESPE/NA36198	Nanohybrid composite resin	Bis‐GMA, UDMA, Bis‐EMA, TEGDEMA, zirconia/silica fillers	Apply resin composite in increments of 1.5 mm, light‐cure for 40 s each increment

Abbreviations: Bis‐EMA, ethoxylated bisphenol A‐glycidyl methacrylate; Bis‐GMA, bisphenol A‐glycidyl methacrylate; HEMA, hydroxyethyl methacrylate; MDP, methacryloyloxydecyl dihydrogen phosphate; TEGDMA, triethylene glycol dimethacrylate; UDMA, urethane dimethacrylate.

### 2.3 Sample Preparation

One hundred and eight maxillary single‐rooted premolars, from young patients (18–25 years old), extracted for orthodontic reasons, are selected. The teeth are intact with no defect, fracture, or crack lines as verified under ×20 magnification. The samples are cleaned and disinfected in a 0.5% chloramine solution and then stored in distilled water at 4°C. The selected teeth had similar size, measured with a digital caliper (Mitutoyo Digimatic; Mitutoyo, Kawasaki, Japan). Before embedding the teeth in a cylinder of self‐curing acrylic resin up to 1 mm below the cementoenamel junction (CEJ), their roots are covered with a 0.2–0.3 mm layer of melted wax. This layer is replaced with a polyether impression material to mimic the periodontal ligament. The long axis of the tooth is perpendicular to the base of the cylinder (Shafiei et al. 2019b). The teeth are randomly allocated into 9 groups (*n* = 12).

#### 2.3.1 Group 1 (ST)

The sound/intact teeth with no cavity preparation served as a negative control.

In groups 2–9, standardized MOD cavities were prepared with the gingival margin placed 1 mm coronal to the CEJ, using a diamond bur (5836.FG.014, Komet, Rock Hill, SC, USA) with a high‐speed handpiece under water spray. The cavity dimensions were occlusal width = 2/3 of intercuspal width; pulpal depth = 2.5 mm; proximal box width = 1/2 of buccolingual dimensions; axial width = 2 mm; and axial depth = 1.5 mm. The facial and palatal walls of the cavities were parallel to the long axis of the teeth (Shafiei et al. 2019a). The Cavo surface margins were prepared at 90° with rounded internal line angles. One experienced operator performed all preparatory procedures. Measurements were made with a digital caliper (Mitutoyo Digimatic; Mitutoyo, Kawasaki, Japan) with 0.1 mm sensitivity for proper and accurate standardization of cavity dimensions. The dental bur was replaced after every five preparations.

#### 2.3.2 Group 2 (Non‐Res)

The prepared teeth were not restored and used as a positive control.

#### 2.3.3 Group 3, Selective Enamel Etch (SEE)

The enamel margins were etched with 37% phosphoric acid for 15 s. After rinsing and drying the cavity, two separate coats of All‐Bond Universal (ABU; Bisco Inc., Schaumburg, IL, USA) were applied and scrubbed on the preparation with a micro brush for 10–15 s per coat without light curing between coats. Excess solvent was evaporated by thoroughly air‐drying with an air syringe for 10 s and light‐cured for 10 s. An LED curing unit (Bluephase, Ivoclar Vivadent, Schaan, Liechtenstein), with a light intensity greater than 1000 mW/cm^2^, was used throughout the study. The intensity of the curing unit is determined by using a dental radiometer before the unit is used.

#### 2.3.4 Group 4, Etch‐and‐Rinse (E&R)

Enamel and dentin were etched with 37% phosphoric acid for 15 s, after rinsing and drying the cavity, ABU applied, and light cured as described in group SEE.

#### 2.3.5 Group 5, Selective Dentin Etch (SDE)

The enamel margins were etched with 37% phosphoric acid for 15 s, and in the last 3–5 s, phosphoric acid was applied to the dentin surface. After rinsing and drying the cavity, ABU was applied and light‐cured.

#### 2.3.6 Group 6, Selective Enamel Etch + Resin Layer (See + AHA)

The bonding procedure was the same as the SEE group, except that a HEMA‐free bonding resin (porcelain bonding resin, Bisco, USA) was applied on the cured ABU for 20 s. Adhesive thinning was gently performed for 5 s and light‐cured for 10 s.

#### 2.3.7 Group 7, Etch‐and‐Rinse + Resin Layer (E&R + AHA)

The bonding procedure was the same as the E&R group, except that the same resin layer was applied on the cured ABU, similar to the previous group.

#### 2.3.8 Group 8, Selective Dentin Etch + Resin Layer, With Pre‐Curing (SDE + AHA/C)

The bonding procedure was the same as the SDE group, except that the same resin layer was applied on the cured ABU, like the previous group.

#### 2.3.9 Group 9, Selective Dentin Etch + Resin Layer With Co‐Curing (SDE + AHA/NC)

The bonding procedure was the same as group SDE + AHA/C, except that ABU was used without being separately light‐cured and co‐cured with the resin layer instead.

The cavities were restored with a nanohybrid resin composite (Filtek Z250 XT, 3 M ESPE) using an incremental technique, using the Tofflemire matrix system. Photopolymerization was done for 40 s with each increment. After completing the restoration, the matrix band and retainer were removed, and additional light‐curing was done directly at each proximal area for 40 s. The excess material was carefully removed with abrasive discs (Sof‐Lex, 3 M ESPE). All teeth were kept in distilled water at 37°C for 24 h. After storage, all the specimens were subjected to 100,000 cycles of application of 50 N loading forces at a frequency of 0.5 Hz in a mastication simulation machine (Chewing Stimulator CS4; SD Mechatronic, Feldkirchen, Westerham, Germany). The mechanical load was applied to the center of the occlusal surface in contact with both cusp ridges using a stainless‐steel antagonist with a rounded end that was 6 mm in diameter in a water environment (Serin Kalay et al. 2016). After a 6‐month water storage period and thermal cycling (Vafaie Inc, Tehran, Iran) for 1000 cycles at 5°C/55°C (dwell time: 15 s) (Shafiei et al. 2019b).

### 2.4 Fracture Resistance Test

**Figure 1:**
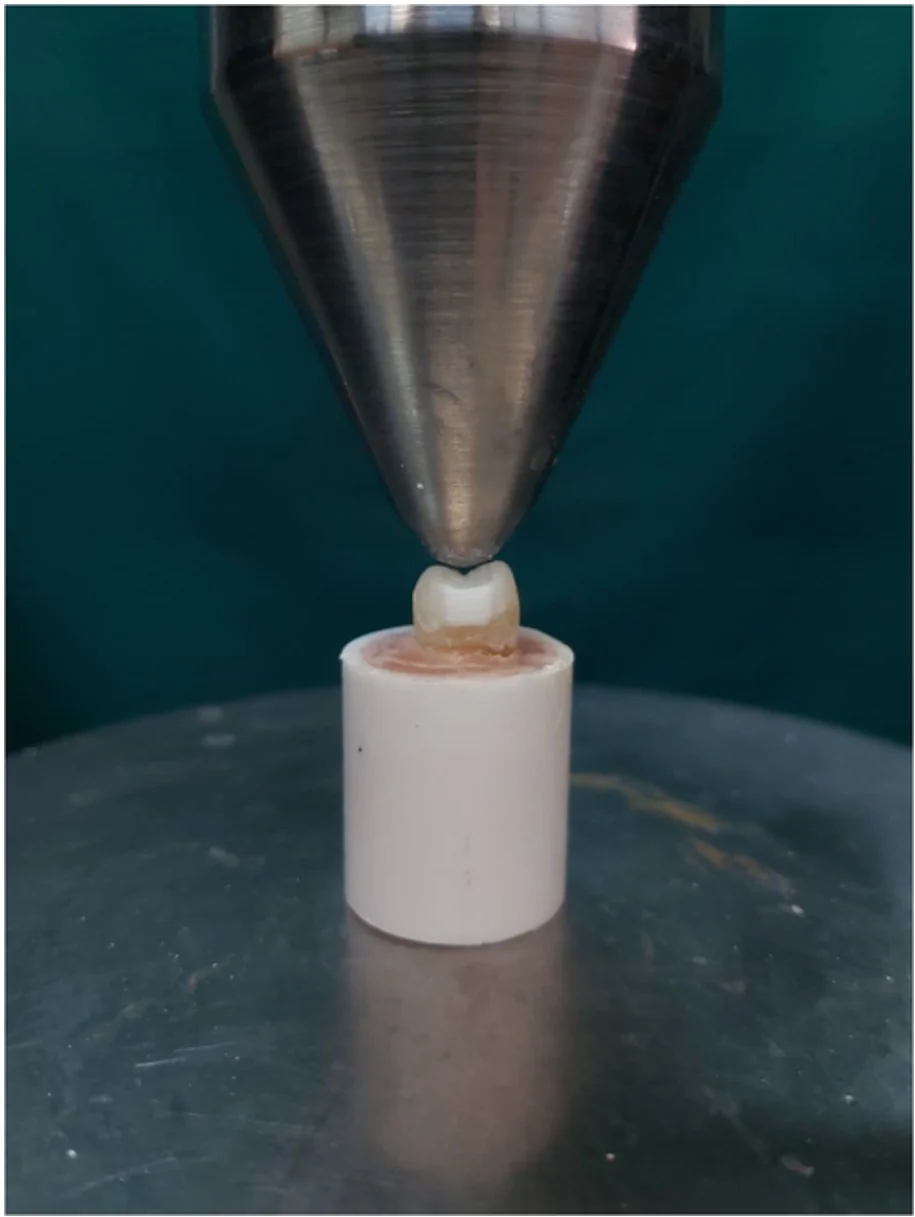
Representative specimen mounted in the universal testing machine for fracture resistance testing.

**Figure 2:**
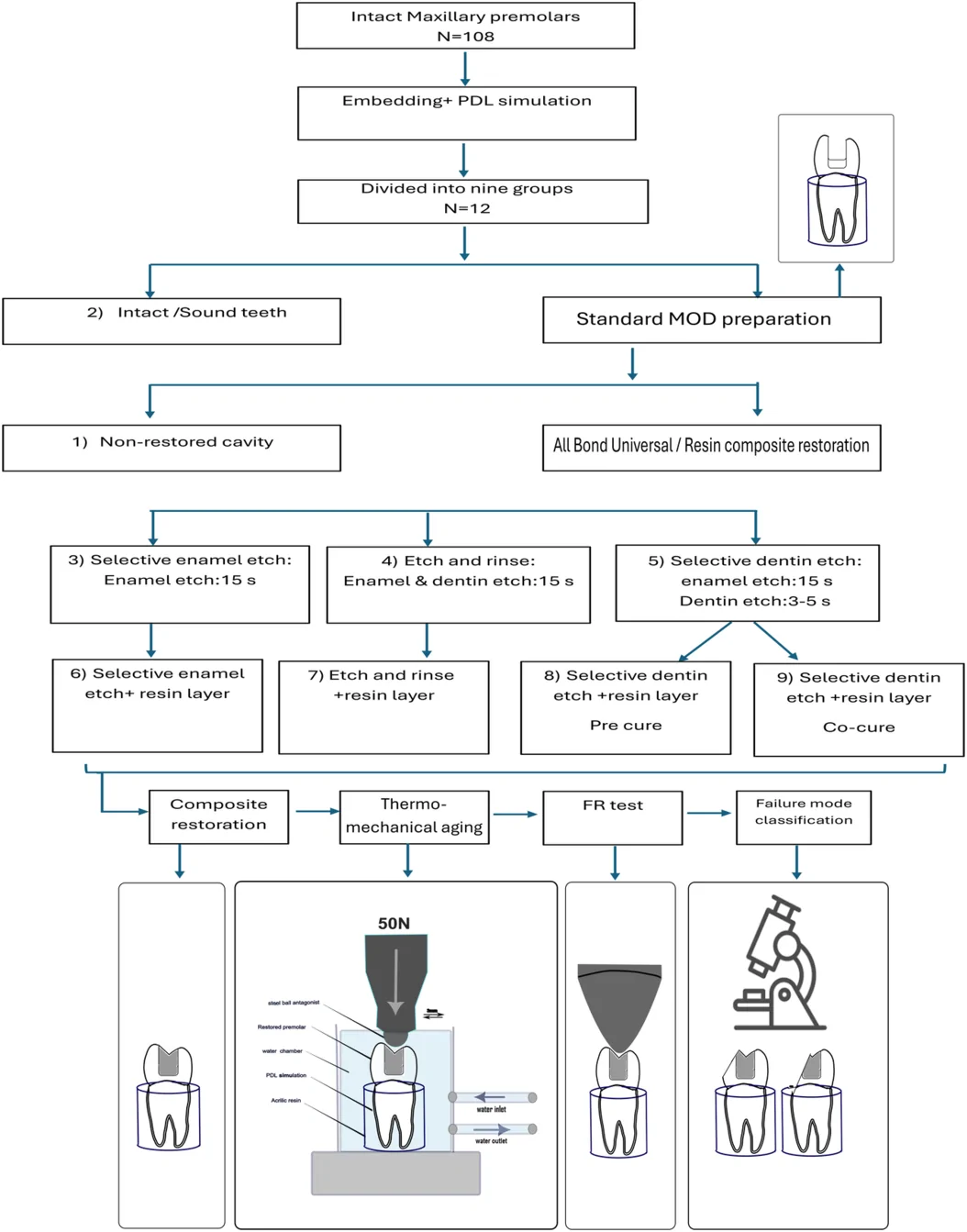
Schematic representation of the study design. The diagram illustrates specimen preparation, PDL simulation, standardized MOD cavity preparation, allocation into nine experimental groups according to the surface treatment and adhesive protocol, composite restoration, thermo‐mechanical aging, fracture resistance testing, and failure mode analysis.

The specimens were subjected to a compressive load at a crosshead speed of 1 mm/min in a universal testing machine (Instron Z020, Zwick Roell, Ulm, Germany). The compressive load was applied parallel to the long axis of the tooth with a 6 mm diameter stainless steel antagonist placed in the center of the tooth with contacts only on the buccal and palatal cuspal inclines (Figure 1). The peak force required for fracture was recorded in newtons as the fracture strength (FR) value. A schematic representation of the study design is shown in Figure 2.

### 2.5 Failure Mode Analysis

Under a stereomicroscope, failure patterns were classified as restorable or unrestorable based on whether the fracture line extended beyond the cementoenamel junction (Shafiei et al. 2019a).

### 2.6 Statistical Analysis

After confirmation of normal distribution of FR data using the Shapiro–Wilk test, one‐way ANOVA was applied to analyze differences among the nine groups, followed by Tukey's post hoc test for pairwise comparison (*p* < 0.05). Statistical analyses were computed using SPSS version 26 software (SPSS Inc., USA).

## 3 Results

The Buccopalatal and mesiodistal dimensions of the used teeth were 7 and 9 mm, respectively, with a variation of 0.5 mm for each dimension.

**Figure 3:**
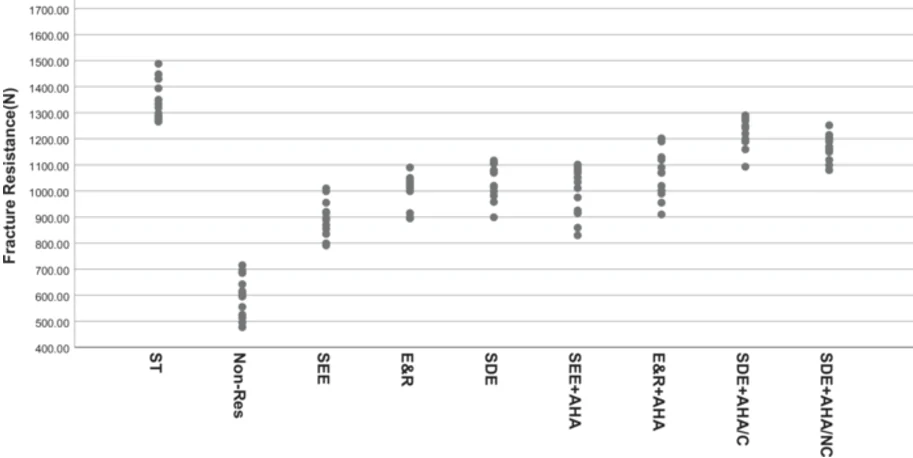
Scatter plot illustrating the distribution of fracture resistance (*N*) values among the experimental groups. Each dot represents one specimen.

Mean fracture load values in newtons (*N*) for the nine tested groups are shown in Table 2. The distribution of fracture resistance values for individual specimens in each experimental group is presented as a scatter plot in Figure 3.

**Table 2:** Fracture load in newtons (mean ± standard deviation) for nine study groups (*n* = 12).

	Group code	Group description	mean ± SD
1	ST	Sound/intact tooth	1347.16 (75.70)^A^
2	Non‐res	Prepared/not restored	593.25 (81.11)^B^
3	SEE	Selective enamel etching,15 s	894.50 (70.41)^C^
4	E&R	Etch‐and‐rinse, enamel and dentin,15 s	988.17 (68.50)^CD^
5	SDE	Selective dentin etching for 3–5 s	1038.75 (70.78)^D^
6	SEE + AHA	Selective enamel etching+ resin layer	995.17 (93.20)^D^
7	E&R + AHA	Etch‐and‐rinse + resin layer	1062.41 (90.44)^DE^
8	SDE + AHA/C	SDE+ resin layer/pre‐curing	1222.00 (58.70)^F^
9	SDE + AHA/NC	SDE+ resin layer/Co‐curing	1166.50 (50.30)^EF^

*Note:* Same uppercase letters indicate no significant difference in fracture loads among groups (*p* < 0.05).

According to one‐way ANOVA, there was a significant difference between groups (*p* < 0.001). The sound teeth group demonstrated the highest load to fracture, and the non‐res group presented the lowest fracture strength value. Both groups significantly differed from the others (*p* < 0.001). All restored groups significantly improved FR values compared with the non‐res group (*p* < 0.001). However, their values were significantly lower than sound teeth.

Among the restored groups, the highest FR was detected in SDE + AHA/C group (1222.0 ± 58.70 N), which was significantly higher than the others (*p* < 0.001) except SDE + AHA/NC group (1166.50 ± 50.30 N) (*p* = 0.664). This indicated a high FR performance of the combination of SDE and resin layer.

SDE revealed a significantly higher FR (1038.75 ± 70.78 N) than SEE group (*p* < 0.001), but did not differ from E&R group (*p* = 0.766), SEE group did not differ significantly from E&R group (894.50 ± 70.41 N vs. 988.17 ± 68.50 N) (*p* = 0.063).

Resin layer significantly improved FR of SEE (*p* = 0.033), this effect was not detected in E&R group (*p* = 0.272). However, the two later groups had comparable FR. Also, resin layer significantly increased FR in SDE in both prior curing and co‐curing groups (*p* < 0.001, *p* = 0.002, respectively).

**Figure 4:**
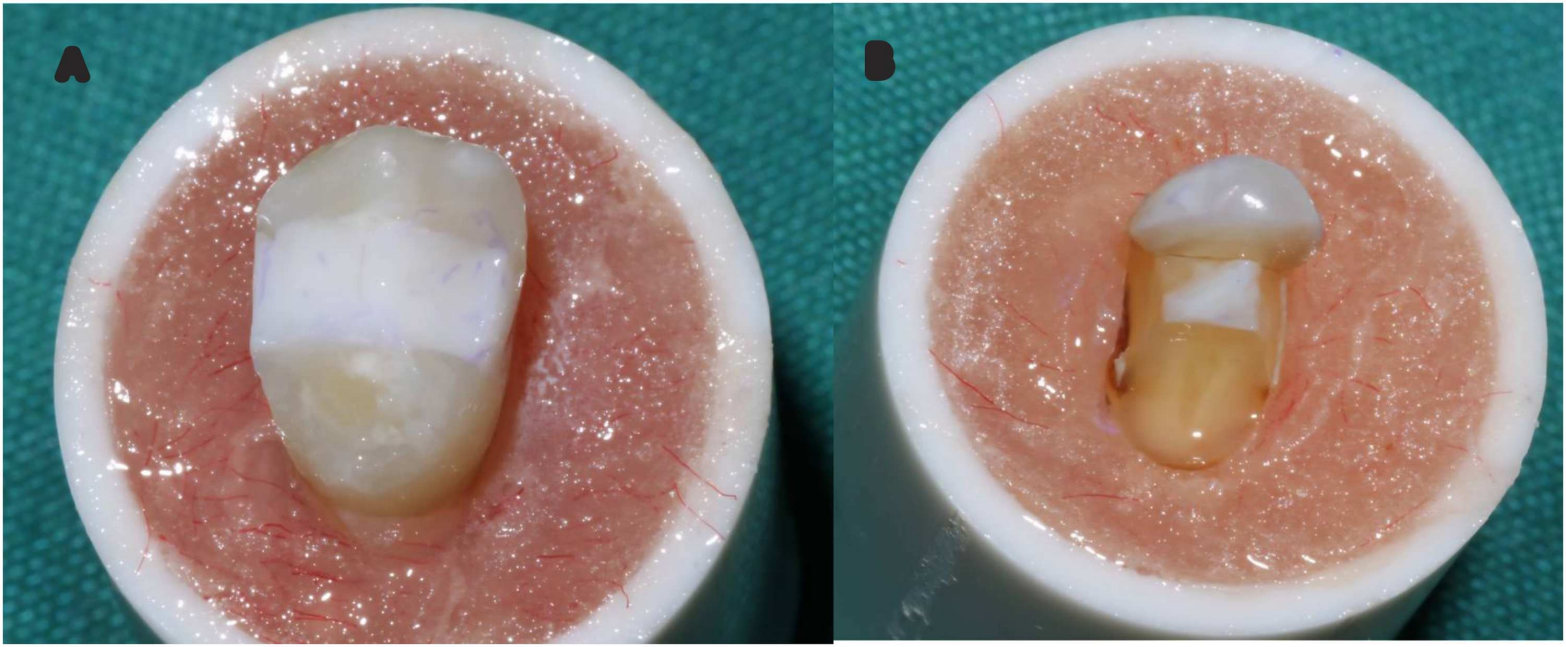
Representative fracture patterns of MOD resin composite–restored premolars after compressive loading: (A) Restorable fracture, in which the tooth structure remains repairable. (B) Unrestorable fracture, in which the damage is catastrophic and not suitable for repair.

Figure 4 shows representative fracture patterns of resin composite–restored premolars after the loading test, including restorable and unrestorable fracture patterns. Assessment of the distribution of fracture patterns, as shown in Table 3, revealed that adding a resin layer to the SEE, E&R, and especially the SDE groups, with or without ABU pre‐curing, resulted in most failures being desirable and repairable.

**Table 3:** Distribution of fracture patterns of nine experimental groups (*n* = 12).

		Fracture pattern	
Groups		Restorable	Unrestorable
1	ST	12 (100%)	0
2	Non‐res	5 (41/6%)	7 (58/3%)
3	SEE	5 (41/6%)	7 (58/3%)
4	E&R	5 (41/6%)	7 (58/3%)
5	SDE	7 (58/3%)	5 (41/6%)
6	SEE + AHA	8 (66/66%)	4 (33/33%)
7	E&R + AHA	8 (66/66%)	4 (33/33%)
8	SDE + AHA/C	10 (83/3%)	2 (16/6%)
9	SDE + AHA/NC	10 (83/3%)	2 (16/6%)

## 4 Discussion

This study evaluated the impact of SDE, resin layer, and their combined application in ABU/composite restorations on the strength recovery of premolars with MOD preparations. Previous studies have demonstrated the beneficial effect of SDE on long‐term bond strength of a mild UA (Stape et al. 2022, 2021, 2018). In the mentioned studies, Stape et al. applied 320‐grit SiC paper to standardize the smear layer on the flat dentin surface. This resulted in greater thickness and density of smear layer compared to fine SiC (600‐grit), which was more similar to bur‐cut produced smear layer in a prepared cavity, preventing overestimation of bond strength in SE mode (Stape et al. 2022). Many bond strength studies assess bonding on flat dentin surfaces without the influence of polymerization shrinkage stress from the overlying resin composite in the cavity (Tang et al. 2023). In contrast, FR studies, including the present one, better simulate clinical conditions by applying chewing forces on restored teeth. The cyclic loading and thermocycling used in this study significantly influence the long‐term performance of adhesive systems (Shafiei et al. 2019b). Moreover, a bonded enamel margin has been reported to protect the resin–dentin bond of simplified adhesives against degradation (Gamborgi et al. 2007). This protective effect, along with the simultaneous stability of the enamel and dentin–resin bonds during thermo‐mechanical aging, may have contributed to the FR outcomes of the restored teeth with different ABU application protocols in this study.

In light of our findings, all application protocols of ABU in composite restorations were able to partially recover the premolar's strength. However, this reinforcing effect was not the same across all experimental groups. Therefore, the null hypothesis was rejected. In the present study, the ER mode was compared with the SEE mode, not just the SE one. Since UAs—especially ultra‐mild UAs—are not capable of creating an adequate and durable enamel bond (Saitamon et al. 2024; Takamizawa et al. 2016), enamel acid etching is therefore combined with the SE mode on dentin surfaces of MOD cavities (SEE). These two modes of ABU resulted in comparable outcomes in terms of the FR of the aged, restored premolars. A recent study demonstrated a significantly higher FR for Single Bond Universal (an ultra‐mild UA) when enamel was acid‐etched (ER and SEE) compared to using the SE mode alone on both enamel and dentin (Saitamon et al. 2024). They also reported that, after thermal cycling, a significant decline in FR occurred in the ER mode. Nevertheless, the FR values of the ER and SEE thermal‐aged groups did not differ from each other (Saitamon et al. 2024). These findings are in line with the thermo‐mechanically aged groups in the present study. Systematic reviews reported no significant difference between the ER and SE modes of UAs in terms of immediate or long‐term dentin bond strength (Chen et al. 2022; Kaczor et al. 2018).

Recently, some authors have recommended the SDE protocol using reduced acid etching time to improve the long‐term bonding ability of UAs (Stape et al. 2022, 2021, 2018). Since the demineralizing efficacy of phosphoric acid etching is time‐dependent, this beneficial effect was demonstrated to be due to the selective removal of the smear layer without overexposure of demineralized collagen. The important role of these factors in bond degradation has been documented (Stape et al. 2022, 2021, 2018). Furthermore, SDE is capable of maintaining a higher content of Ca within the hybrid layer, resulting in greater resistance to water aging (Stape et al. 2018). This SDE protocol, along with enamel acid etching used in the current study, resulted in a higher FR compared to the SEE group. However, this did not differ from the ER method. This result indicated the important role of removing the thick smear layer created with the bur used in cavity preparation before ABU application. The ultra‐mild pH of ABU may prevent its penetration through the whole of this smear layer. In agreement with our findings, a recent study revealed no difference between two etching times (15 and 3 s) in terms of thermo‐mechanical aged dentin bond strength of two types of mild UAs (Ismail and Soliman 2025). Recently, the bond strength of two UAs, including ABU, after thermal aging was found to be comparable in three etching modes. Those authors confirmed that different pH and composition of UAS are determining factors in the bonding ability of UAs (Balaban and Arısu 2025). However, these studies were performed on flat dentin prepared with 600‐grit SiC paper (Balaban and Arısu 2025; Ismail and Soliman 2025).

The other modified approach of UAs employed in this study was applying an AHA layer on ABU in different etching modes. The results revealed its significant beneficial effect on FR for the SEE mode and not for the ER mode of ABU. However, both groups had comparable FR. Previous studies reported that some UAs in SE mode could benefit from an AHA layer in terms of aged dentin bond strength. This effect was found to be product‐dependent and etching‐mode‐dependent (Ahmed et al. 2019; Ermis et al. 2019). No study has evaluated the effect of AHA on the strength of UA–restored teeth. The higher immediate and aged bond strength of ABU in SE mode with an AHA layer was demonstrated (Nunes et al. 2005). However, in an earlier study, this increase in immediate bond strength was not significant for ABU (Muñoz et al. 2014). The AHA layer is capable of decreasing the relative concentration of retained solvents and unreacted monomers in the adhesive layer, thereby increasing the mechanical properties of the adhesive layer and the in‐situ degree of conversion, and also decreasing the permeability of the adhesive layer (Ermis et al. 2019; Muñoz et al. 2014; Sezinando et al. 2015). This AHA layer was usually applied on cured UA (Ermis et al. 2019; Muñoz et al. 2014; Sezinando et al. 2015); however, its beneficial effect was also found when some UAs were not separately light‐cured (Ahmed et al. 2019, 2020). Nevertheless, this effect was not observed for ABU and Single Bond Universal (Wang et al. 2023). According to a recent study, the AHA layer applied on UAs with no prior curing as primers in a challenging high C‐factor cavity model revealed reliable and durable bonding effectiveness. This has been attributed to increased hydrophobicity and mechanical properties of the adhesive and adhesive thickness, imparting better absorption/relief of shrinkage stress of overlaid restorative composite (Tang et al. 2023). The results of Ermis et al.'s study on the comparative effect of the AHA layer on cured and uncured UAs in SE mode on their bonding durability revealed the beneficial effect solely in the case of prior curing of two UAs. It was explained that a waterproof and highly hydrophobic interface could seal the adhesive interface, minimizing water uptake and adhesive permeability (Ermis et al. 2019).

In line with the beneficial effect of applying AHA, a manufacturer has recently introduced a new two‐step self‐etch adhesive (G2‐ Bond, GC, Tokyo, Japan). Its primer is a HEMA‐free and moderately acidic primer, and the bonding resin contains hydrophobic monomers with no HEMA or MDP. This hydrophobic character ensures mechanical stability and less degradation against water sorption in the long term (Kitahara et al. 2024; Tichy et al. 2021). Some authors demonstrated that the more hydrophobicity of the bonding resin of G2‐Bond resulted in superior enamel and dentin bond durability than the two conventional two‐step self‐etch adhesives (Takamizawa et al. 2023).

The interesting finding of this study was that the combination of SDE and AHA layer had the greatest reinforcing effect, with a significant difference from the other groups, when the AHA layer was applied on cured ABU. This effect was less (without a significant difference) when AHA was applied to uncured ABU. Recently, Stape et al. concluded that the association of increased hydrophobic monomer content at the hybrid layer and a less‐aggressive ER protocol (3‐second etching) increases fatigue strength of UAs after long‐term aging. This improved the durability of mild UAs. In their study, UA acted as a primer and was left uncured before applying AHA (Stape et al. 2024). This provided an additional 20 s for continued etching during the application of AHA over uncured UA (Stape et al. 2024).

Furthermore, these two combined SDE and AHA groups revealed a higher ratio of restorable fracture (83.3%). This could be advantageous, preserving the tooth following fracture under unexpectedly high loading, allowing for restoration replacement. It seemed that mild dentin etching, combined with a resin layer with low elastic modulus and hardness, contributed to stress absorption and redistribution, thereby facilitating stress relief and reducing the direct transmission of excessive forces to the tooth structures. This beneficial effect was previously indicated when a self‐adhesive resin layer was applied under resin composite restorations (Shafiei et al. 2019a).

In the current study, 3–5 s etching time instead of 3 s was applied in SDE mode because control of this short‐time etching in the whole of the MOD cavity is problematic. Clinically, controlling different etching times separately for enamel (15 s) and dentin (3–5 s) in a cavity may be a problematic issue. Some authors have demonstrated that the use of shortened pre‐etching times for enamel, less than 15 s, improved the bonding performance of single‐step self‐etch adhesives and Single Bond Universal (Tsujimoto et al. 2016). This reduced etching time for both enamel and dentin would be more applicable and simpler clinically. Future research could investigate this simplified, less‐aggressive etching protocol.

The degree of hydrophobicity (presence or absence of HEMA) and thickness of the AHA layer differed in various related studies. These factors may affect the reported results. Some authors recommended using a highly filled, HEMA‐free, hydrophobic adhesive layer, such as the adhesive resin of OptiBond FL, as AHA because of generating a thicker adhesive layer (Ahmed et al. 2020; Sezinando et al. 2015). However, the AHA layer used in this study did not have any nano‐filler particle content. Further studies were suggested to be conducted with more UAs with different pH values and AHA with higher filler content. These factors may have different strengthening effects on the restored teeth after long‐term aging.

The different aging protocols consisted of mechanical loading, thermocycling, and water storage, employed for seven application modes of ABU in resin composite restoration in this study, which could be a strength of the study. A masticatory simulator with 100,000 cycles of loading was employed to mimic the clinical condition that occurs during chewing in at least 1 year of in vivo clinical use. The frequency of used mechanical loading was 0.5 Hz, which is close to the masticatory cycle in an intraoral condition (Serin Kalay et al. 2016). Although a relatively low number of thermal cycles was used in this study, 6‐month water storage was employed. Long‐term water storage was found to remain the preferable aging protocol for the resin‐dentin bonded interface (Stape et al. 2018; Takamizawa et al. 2023). This preference is due to both water movement, hydrolysis of the hydrophilic resin component, and activation of endogenous enzymatic degradation following water storage. This activation might not occur by thermocycling. The repetitive temperature fluctuation during thermocycling may impair the activity of matrix metalloproteinases (Pashley et al. 2004; Stape et al. 2018). These enzymes are responsible for degrading unprotected collagen fibers in the hybrid layer (Pashley et al. 2004).

The present study had some limitations; only one universal adhesive, All‐Bond Universal, and only one type of resin composite was used. Since bonding performance and the effect of an AHA layer may be material‐dependent, the findings cannot be generalized to other universal adhesives and resin composite materials. Also, FR of restored teeth may be influenced by the mechanical properties, filler content, polymerization shrinkage, and elastic modulus of the restorative composite. FR was evaluated under static compressive loading on the restored teeth. In intraoral situations, the restored teeth are submitted to different dynamic functional stresses, such as shear and tensile, in addition to compressive ones. The other in vivo conditions were not closely mimicked, such as pulpal pressure, pH changes, and enzymatic challenges. Further long‐term studies and clinical trials should be conducted to confirm the results of this in vitro study.

## 5 Conclusion

Overall, removal of the smear layer with 3–5 s of etching in SDE could allow ABU to sufficiently interact with dentin, and along with a HEMA‐free hydrophobic resin layer on cured ABU, provided the best reinforcing effect on the strength of the aged resin composite‐restored premolars.

## Author Contributions

**Fereshteh Shafiei:** conceptualization, study design, supervision of laboratory procedures, statistical consultation, data interpretation, manuscript review and editing. **Marzieh Moradian:** conceptualization, study design, data interpretation, manuscript review and editing, correspondence with the journal. **Marzieh Naseri Mojarad:** experimental procedures, data collection, manuscript drafting.

## Conflicts of Interest

The authors declare no conflicts of interest.

## Data Availability

The data that support the findings of this study are available on request from the corresponding author. The data are not publicly available due to privacy or ethical restrictions.
